# A Note on Causation versus Correlation in an Extreme Situation

**DOI:** 10.3390/e23030316

**Published:** 2021-03-07

**Authors:** X. San Liang, Xiu-Qun Yang

**Affiliations:** 1 Nanjing Institute of Meteorology, 219 Ningliu Blvd, Nanjing 210044, China; 2 School of Atmospheric Sciences, Nanjing University, 163 Xianlin Avenue, Nanjing 210023, China; xqyang@nju.edu.cn

**Keywords:** causality, time series, information flow, correlation

## Abstract

Recently, it has been shown that the information flow and causality between two time series can be inferred in a rigorous and quantitative sense, and, besides, the resulting causality can be normalized. A corollary that follows is, in the linear limit, causation implies correlation, while correlation does not imply causation. Now suppose there is an event *A* taking a harmonic form (sine/cosine), and it generates through some process another event *B* so that *B* always lags *A* by a phase of π/2. Here the causality is obviously seen, while by computation the correlation is, however, zero. This apparent contradiction is rooted in the fact that a harmonic system always leaves a single point on the Poincaré section; it does not add information. That is to say, though the absolute information flow from *A* to *B* is zero, i.e., $T_{A\to B}=0$, the total information increase of *B* is also zero, so the normalized $T_{A\to B}$, denoted as $\tau_{A\to B}$, takes the form of $\frac{0}{0}$. By slightly perturbing the system with some noise, solving a stochastic differential equation, and letting the perturbation go to zero, it can be shown that $\tau_{A\to B}$ approaches 100%, just as one would have expected.

## 1. A Review of the Rigorous Information Flow-Based Causality Analysis

Causal inference is a fundamental problem in scientific research. Recently it has been shown that the problem can be recast into the framework of information flow, another fundamental notion in general physics which has wide applications in different disciplines (see [1]), and hence can be put on a rigorous footing. The causality between two time series can then be analyzed in a quantitative sense, and, besides, the resulting formula is very concise in form. In the linear limit, it involves only the usual statistics namely sample covariances [2], making the important and otherwise difficult problem an easy task.

To briefly review the theory, consider a two-dimensional continuous-time stochastic system for state variables $x=(x_{1},x_{2})$

(1) $$\begin{array}{c} \frac{dx}{dt}=F\left(x,t\right)+B\left(x,t\right)\dot{w}, \end{array}$$

where $F=(F_{1},F_{2})$ may be arbitrary nonlinear functions of x and *t*, $\dot{w}$ is a vector of white noise, and $B=(b_{ij})$ is the matrix of perturbation amplitudes which may also be any functions of x and *t*. Here we adopt the convention in physics and do not distinguish deterministic and random variables; in probability theory, they are ususally distinguished with capital and lower-case symbols. Assume that F and B are both differentiable with respect to x and *t*. Then the information flow from $x_{2}$ to $x_{1}$ (in nats per unit time) can be explicitly found in a closed form [3] (the multiple-dimensional case is referred to [1]):

(2) $$\begin{array}{c} T_{2\to 1}=-E\left[\frac{1}{\rho_{1}}\frac{\partial (F_{1}\rho_{1})}{\partial x_{1}}\right]+\frac{1}{2}E\left[\frac{1}{\rho_{1}}\frac{\partial^{2}g_{11}\rho_{1}}{\partial x_{1}^{2}}\right], \end{array}$$

where *E* stands for mathematical expectation, and $g_{ii}=\sum_{k=1}^{n}b_{ik}b_{ik}$, $\rho_{i}=\rho_{i}\left(x_{i}\right)$ is the marginal probability density function (pdf) of $x_{i}$. The rate of information flowing from $x_{1}$ to $x_{2}$ can be obtained by switching the indices. If $T_{j\to i}=0$, then $x_{j}$ is not causal to $x_{i}$; otherwise it is causal, and the absolute value measures the magnitude of the causality from $x_{j}$ to $x_{i}$. For discrete-time mappings, the information flow is in much more complicated a form; see [1].

In the case with only two time series (no dynamical system is given) $X_{1}$ and $X_{2}$, under the assumption of a linear model with additive noise, the maximum likelihood estimator (MLE) of the rate of information flowing from $X_{2}$ to $X_{1}$ is [2]

(3) $$\begin{array}{c} {\hat{T}}_{2\to 1}=\frac{C_{11}C_{12}C_{2,d1}-C_{12}^{2}C_{1,d1}}{C_{11}^{2}C_{22}-C_{11}C_{12}^{2}}, \end{array}$$

where $C_{ij}$ is the sample covariance between $X_{i}$ and $X_{j}$, and $C_{i,dj}$ the sample covariance between $X_{i}$ and a series derived from $X_{j}$ using the Euler forward differencing scheme (also see the Euler–Maruyama scheme in [4]): ${\dot{X}}_{j,n}=\left(X_{j,n+k}-X_{j,n}\right)/\left(k\Delta t\right)$, with k≥1 some integer. Note that Equation (3) is rather concise in form; it only involves the common statistics, i.e., sample covariances. In other words, a combination of some sample convariances will give a quantiative measure of the causality between the time series. This makes causality analysis, which otherwise would be complicated with the classical empirical/half-empirical methods, very easy. Nonetheless, note that Equation (3) cannot replace (1); it is just the mle of the latter. Statistical significance test must be performed before a causal inference is made based on the computed $T_{2\to 1}$. For details, refer to [2].

Considering the long-standing debate ever since Berkeley (1710) [5] over correlation versus causation, we may rewrite (3) in terms of linear correlation coefficients, which immediately implies [2]:

*Causation implies correlation, but correlation does not imply causation.*

The above formalism has been validated with many benchmark systems (e.g., [1]) such as baker transformation, Hénon map, Kaplan-Yorke map, Rössler system, etc. It also has been successfully applied to the studies of many real world problems such those in financial economics (e.g., the “Seven Dwarfs vs. a Giant” problem [6]), earth system science (e.g., the Antarctica mass balance problem [7] and the global warming problem [8]), neuroscience (e.g., the concussion problem [9]), to name but a few.

## 2. The Question

Now suppose we have a dynamic event *A* which drives another event *B*. The former has a harmonic form, leading the latter by a phase of π/2. That is to say, the time series, say, $\left\{x_{A}\left(t\right)\right\}$ and $\left\{x_{B}\left(t\right)\right\}$ resulting from the two, are in quadrature. Then the correlation between the two is zero. Here by zero-correlation we mean a zero integral

$$\begin{array}{c} \int_{\Omega }\left(x_{A}\left(t\right)-{\bar{x}}_{A}\right)\left(x_{B}\left(t\right)-{\bar{x}}_{B}\right)dt, \end{array}$$

with the integration domain Ω being one period or more periods, and the overbar being the mean over the domain. However, since *A* causes *B*, the result is apparently in contradiction to the above corollary that “causation implies correlation”.

## 3. The Solution

The problem can be more formally stated with the harmonic system:

(4) $$\frac{dx}{dt}=F\left(x,t\right)=Ax=\left[\begin{array}{cc} a_{11} & a_{12}\\ a_{21} & a_{22} \end{array}\right]\left[\begin{array}{c} x_{1}\\ x_{2} \end{array}\right]=\left[\begin{array}{cc} 0 & -1\\ 1 & 0 \end{array}\right]\left[\begin{array}{c} x_{1}\\ x_{2} \end{array}\right].$$

If the system is initialized with $x_{1}\left(0\right)=1$, ${\dot{x}}_{1}\left(0\right)=1$, the solution is, $x_{1}=\cos t$, $x_{2}=\sin t$. Thus, the population covariance $\sigma_{12}=\int_{\Omega }\cos t\sin tdt=0$ (Ω is one period or many periods). This yields an information flow from $x_{2}$ to $x_{1}$:

(5) $$\begin{array}{c} T_{2\to 1}=a_{12}\frac{\sigma_{12}}{\sigma_{11}}=0. \end{array}$$

Fundamentally the above problem arises from the fact that it is a deterministic system. In Granger causality test [10] (also see a recent reference [11]), this case has been explicitly excluded, as in such case the trajectories do not form appropriate ensembles in the sample space. For a harmonic series, it shows on a Poincaré section only one single point; so the total information does not accrue. If the total information does not change, the information flow to $x_{1}$ must also vanish. However, the vanishing information flow does not mean that there is no influence of $x_{2}$ on $x_{1}$. As we argued in Liang (2015), the so-obtained information must be normalized, just as covariance needs to be normalized into correlation, for one to assess the causal influence. Here if the normalizer is zero, $T_{2\to 1}$ involves the indeterminate form $\frac{0}{0}$. We may then approach it by taking the limit. Specifically, we may approach it by enlarging the sample space slightly, i.e., by adding some stochasticity to the system, then take the limit by letting the stochastic perturbation amplitude go zero.

By Liang (2015), the normalizer for $T_{2\to 1}$ is

(6) $$\begin{array}{c} Z_{2\to 1}=|T_{2\to 1}\left|+\right|\frac{dH_{1}^{*}}{dt}\left|+\right|\frac{dH_{1}^{\mathrm{noise}}}{dt}|, \end{array}$$

where on the right hand side, the second term is the contribution from $x_{1}$ itself, and the third term the contribution from noise. In Liang (2015), it has shown that $\frac{dH_{1}^{*}}{dt}$ is a Lyapunov exponent-like, phase-space stretching rate, and $\frac{dH_{1}^{noise}}{dt}$ a noise-to-signal ratio. In this problem, we do not have noise taken into account. However, in reality, noise is ubiquitous. We may hence view a deterministic system as a limit or extreme case as the amplitude of stochastic perturbation goes to zero. For this case, we add to (4) a stochastic term:

(7) $$\begin{array}{c} \frac{dx}{dt}=Ax+B\dot{w}, \end{array}$$

where w is a vector of standard Wiener processes. For simplicity, let the perturbation amplitude B a constant matrix. Further let $G=BB^{T}$, with elements

$$\left(g_{ij}\right)=\sum_{k=1}^{2}b_{ik}b_{jk}.$$

Liang (2008) established that

(8) $$\begin{array}{ccc} & & \frac{dH_{1}^{*}}{dt}=a_{11}=0, \end{array}$$

(9) $$\begin{array}{ccc} & & \frac{dH_{1}^{\mathrm{noise}}}{dt}=\frac{1}{2}\frac{g_{11}}{\sigma_{11}}. \end{array}$$

So in this case, the normalized flow from $x_{2}$ to $x_{1}$ is

(10) $$\begin{array}{c} \tau_{2\to 1}=\frac{a_{12}\frac{\sigma_{12}}{\sigma_{11}}}{|a_{12}\frac{\sigma_{12}}{\sigma_{11}}\left|\;+\;0\;+\;\right|\frac{1}{2}\frac{g_{11}}{\sigma_{11}}|}=\frac{-\sigma_{12}}{|\sigma_{12}|\;+\;\frac{g_{11}}{2}}. \end{array}$$

Likewise,

(11) $$\begin{array}{c} \tau_{1\to 2}=\frac{a_{21}\frac{\sigma_{12}}{\sigma_{22}}}{|a_{21}\frac{\sigma_{12}}{\sigma_{22}}\left|\;+\;\right|a_{22}\left|\;+\;\right|\frac{1}{2}\frac{g_{22}}{\sigma_{22}}|}=\frac{+\sigma_{12}}{|\sigma_{12}|\;+\;\frac{g_{22}}{2}}. \end{array}$$

Note that $\tau_{2\to 1}$ (or $\tau_{1\to 2}$) may be positive or negative. In causal inference, this does not matter; we need only consider the absolute value, although the sign does carry a meaning according to the original formulation. (A positive $\tau_{2\to 1}$ means $x_{2}$ causes the marginal entropy of $x_{1}$ to grow and vice versa; see [1,2].)

Now for the stochastic equation, the covariance matrix Σ evolves as

(12) $$\begin{array}{c} \frac{d\Sigma }{dt}=A\Sigma +\Sigma A^{T}+BB^{T}=A\Sigma +\Sigma A^{T}+G \end{array}$$

Expanding, this is

$$\frac{d\;}{dt}\left[\begin{array}{cc} \sigma_{11} & \sigma_{12}\\ \sigma_{12} & \sigma_{22} \end{array}\right]\;=\;\left[\begin{array}{cc} -\sigma_{12} & -\sigma_{22}\\ \sigma_{11} & \sigma_{12} \end{array}\right]\;+\;\left[\begin{array}{cc} -\sigma_{12} & \sigma_{11}\\ -\sigma_{22} & \sigma_{12} \end{array}\right]\;+\;\left[\begin{array}{cc} g_{11} & g_{12}\\ g_{12} & g_{22} \end{array}\right].$$

We hence obtain the following equation set:

$$\begin{array}{ccc} & & \frac{d\sigma_{11}}{dt}=-2\sigma_{12}+g_{11},\\ & & \frac{d\sigma_{12}}{dt}=-\sigma_{22}+\sigma_{11}+g_{12},\\ & & \frac{d\sigma_{22}}{dt}=2\sigma_{12}+g_{22}. \end{array}$$

Solving, we get

$$\begin{array}{c} \frac{d^{2}\sigma_{12}}{dt^{2}}=-4\sigma_{12}+\left(g_{11}-g_{22}+g_{12}\right). \end{array}$$

So the solution is

$$\begin{array}{c} \sigma_{12}=C_{1}\cos2t+C_{2}\sin2t+\frac{1}{2}\left(g_{11}-g_{22}+g_{12}\right)t^{2}. \end{array}$$

If $\sigma_{12}\left(0\right)=0$, ${\dot{\sigma }}_{12}\left(0\right)=0$, then the integration constants $C_{1}=C_{2}=0$. So

$$\begin{array}{c} \tau_{2\to 1}=\frac{-\sigma_{12}}{|\sigma_{12}|\;+\;\frac{1}{2}g_{11}}=\frac{-1}{1+\frac{g_{11}}{\left(g_{11}-g_{22}+g_{12}\right)t^{2}}} \end{array}$$

Two cases are distinguished:

Case I $g_{12}-g_{22}=\mathrm{const}\neq 0$.
  $$\lim_{g_{11}\to 0}\tau_{2\to 1}=-1.$$
Case II $g_{12}-g_{22}=0$.
  $$\lim_{g_{11}\to 0}\tau_{2\to 1}=\frac{-1}{1+1/t^{2}}.$$
  As *t* goes to infinity, $\tau_{2\to 1}$ also approaches −1.

If initially there exists some covariance, say, $\sigma_{12}\left(0\right)=c$, then $C_{1}=c$, and hence

$$\begin{array}{c} \tau_{2\to 1}=\frac{-1}{1+\frac{g_{11}}{2c\cos2t+\left(g_{11}-g_{22}+g_{12}\right)t^{2}}}. \end{array}$$

In this case, as $g_{11}\to 0$, we always have $\tau_{2\to 1}\to -1$. Either way, the relative information flow $\tau_{2\to 1}$ approaches −1 in the limit of deterministic system.

In the other direction, we now need to consider the uncertainty growth of $x_{2}$ and hence perturb $g_{22}$. Repeating the above procedure, when $\sigma_{12}\left(0\right)={\dot{\sigma }}_{12}\left(0\right)=0$, the normalized information flow is

$$\begin{array}{c} \tau_{1\to 2}=\frac{+\sigma_{12}}{|\sigma_{12}|\;+\;\frac{1}{2}g_{22}}=\frac{+1}{1+\frac{g_{22}}{\left(g_{11}-g_{22}+g_{12}\right)t^{2}}}. \end{array}$$

If $g_{11}+g_{12}=\mathrm{const}\neq 0$, then

$$\begin{array}{c} \lim_{g_{22}\to 0}\tau_{1\to 2}=+1; \end{array}$$

else ($g_{11}+g_{12}=0$)

$$\begin{array}{c} \lim_{g_{22}\to 0}\tau_{1\to 2}=\frac{1}{1-1/t^{2}} \end{array}$$

which approaches to 1 for enough long time (t→∞). On the other hand, if initially there exists some covariance such that $\sigma_{12}\left(0\right)=c$ then

$$\begin{array}{c} \tau_{12}=\frac{1}{1+\frac{g_{22}}{2c\cos2t+\left(g_{11}-g_{22}+g_{12}\right)t^{2}}} \end{array}$$

which implies

$$\begin{array}{c} \lim_{g_{22}\to 0}\tau_{1\to 2}=1. \end{array}$$

This is indeed what we expect. So even for this extreme case, there is no contradiction at all for causal inference using information flow.

## 4. Discussion

To summarize, a recent rigorously formulated causality analysis asserts that, in the linear limit, causation implies correlation, while correlation does not necessarily mean causation. In this short note, an extreme case which apparently contradicts to the assertion is examined. In this case an event $x_{1}$ takes a harmonic form (sine/cosine), and generates through some process another event $x_{2}$ so that $x_{2}$ is always out of phase with $x_{1}$, i.e., lag $x_{1}$ by π/2. Obviously $x_{1}$ causes $x_{2}$, but by computation the correlation between $x_{1}$ and $x_{2}$ is zero. In this study we show that this is an extreme case, with only one point in the phase space and hence the problem becomes singular. We re-examine the problem by enlarging the ensemble space slightly through adding some noise. A stochastic differential equation is then solved for the corresponding covariances, which allows us to obtaint the information flows for the perturbed system. Then as the noisy perturbation goes to zero, the normalized information flow rate from $x_{1}$ to $x_{2}$ is established to be 100%, just as one would have expected. So actually no contradiction exists. (see [12] for how a stochastic differential equation is solved by perturbing it with noise [12].)

One thing that merits mentioning is that, here although it seems that $x_{1}$ causes $x_{2}$, actually here the normalized information flow rate from $x_{2}$ to $x_{1}$ is also 100%. That is to say, for such a harmonic system with circular cause-effect relation, it is actually impossible to differentiate causality by simply assessing which takes place first; anyhow, taking lead by π/2 is equivalent to lagging by 3π/2. The moral is, for a process that is nonsequential (e.g., that in the nonsequential stochastic control systems), circular cause and consequence coexist, it is essentially impossible to distinguish a delay from an advance.
